# Supplementary material for: Selective encoding of priors for flexible categorization but not Bayesian inference in the frontal eye field
Source: bioRxiv. 2026 Apr 20:2024.12.31.630950. Preprint. [Version 2] doi: 10.1101/2024.12.31.630950 (PMC13131809; doi:10.1101/2024.12.31.630950)
Supplement: 1 [file NIHPP2024.12.31.630950V2-supplement-1.pdf]

bioRxiv preprint doi: https://doi.org/10.1101/2024.12.31.630950; this version posted April 20, 2026. The copyright holder has placed this preprint (which was not certified by peer review) in the Public Domain. It is no longer restricted by copyright. Anyone can legally share, reuse, remix, or adapt this material for any purpose without crediting the original authors.

Supplemental information

bioRxiv preprint doi: https://doi.org/10.1101/2024.12.31.630950; this version posted April 20, 2026. The copyright holder has placed this preprint (which was not certified by peer review) in the Public Domain. It is no longer restricted by copyright. Anyone can legally share, reuse, remix, or adapt this material for any purpose without crediting the original authors.

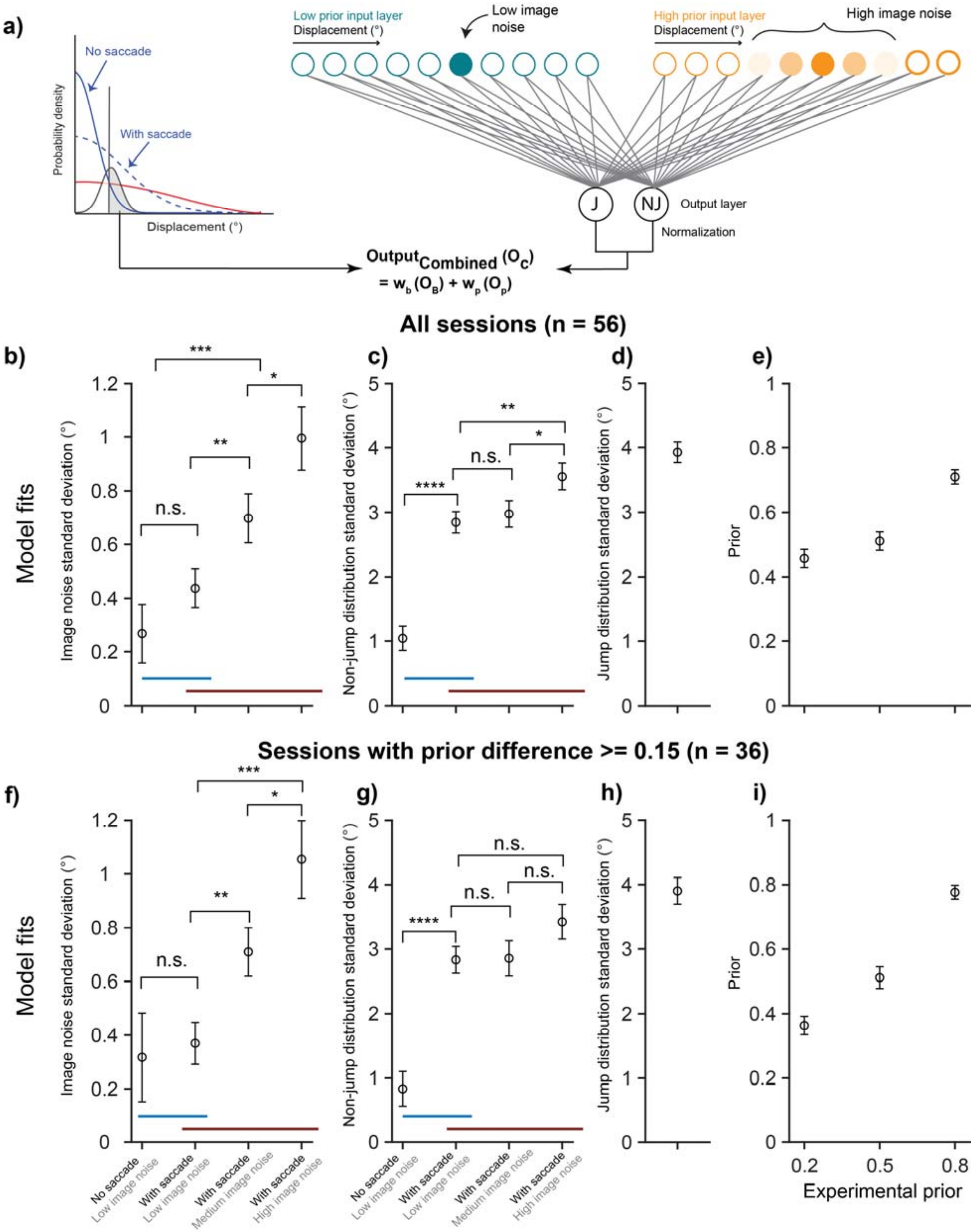

1029

1030 **Figure S1: Combined model parameters fit to behavior.** a) Schematic of the combined
1031 model (duplicated from Fig. 2d). Image noise is parametrized as the width of the distribution

bioRxiv preprint doi: https://doi.org/10.1101/2024.12.31.630950; this version posted April 20, 2026. The copyright holder has placed this preprint (which was not certified by peer review) in the Public Domain. It is no longer restricted by copyright. Anyone can legally share, reuse, remix, or adapt this material for any purpose without crediting the original authors.

centered on the probe displacement and saccade noise is parametrized as the width of the "non-jump" displacement distribution centered on 0. Additionally, we also allowed the "jump" distribution and 3 levels of priors to vary as free parameters. Note that the two conditions with low image noise (with and no saccade) also included a 0.5 prior condition as in Subramanian et al., 2023. The data are not shown in this manuscript to maintain the same prior comparison between the 0.8 and 0.2 conditions across all noise levels, but those trials were included for model fitting. We fixed the weight of the Bayesian component at 0.1 as in the simulations. We fit the model separately to data from each session using maximum likelihood estimation. To simulate the learned weights between the displacement inputs and the "jump" and "non-jump" outputs for each prior condition, we used the value of the psychometric curve fit to all data in that prior condition for each session. b-e) Means and standard errors of fits across all sessions. The image noise parameter varies primarily across the image noise conditions ($F(2) = 10.8$; $p = 5.2169e-05$ on a repeated-measures ANOVA) but does not differ significantly between the no-saccade and common conditions (b). The model fits for the saccade noise parameter (i.e., the width of the non-jump distribution) vary significantly between the no-saccade and common conditions ($p = 2.9514e-10$ on a paired t-test; Cohen's $d = -1.3459$) but much less across the image noise conditions (high image noise vs. low Cohen's $d = 0.51$; high image noise vs medium Cohen's $d = 0.38$; panel (c)). The model fits for the jump distribution (d) and priors (e) were consistent with expectations. f – i) Model fits for the priors themselves may trade off with the noise parameters, which primarily serve to modulate prior use in our model. To test this possibility, we evaluated model fits only for the sessions in which the fits for the low and high prior differed by at least 0.15 ($n = 36$ sessions). Indeed, for these sessions, there was a complete statistical dissociation between the saccade and image noise experiments. There was no significant difference between the image noise fits in the saccade experiment and significant variation across the image noise conditions ($F(2) = 12.19$; $p = 2.8583e-05$; (f)). Saccade noise fits varied significantly across the saccade noise conditions ($p = 6.8431e-07$) but not across the image noise conditions ($F(2) = 2.28$; $p = 0.11$; (g)). The model fits for the jump distribution (h) and priors (i) were once again consistent with expectations.

bioRxiv preprint doi: https://doi.org/10.1101/2024.12.31.630950; this version posted April 20, 2026. The copyright holder has placed this preprint (which was not certified by peer review) in the Public Domain. It is no longer restricted by copyright. Anyone can legally share, reuse, remix, or adapt this material for any purpose without crediting the original authors.

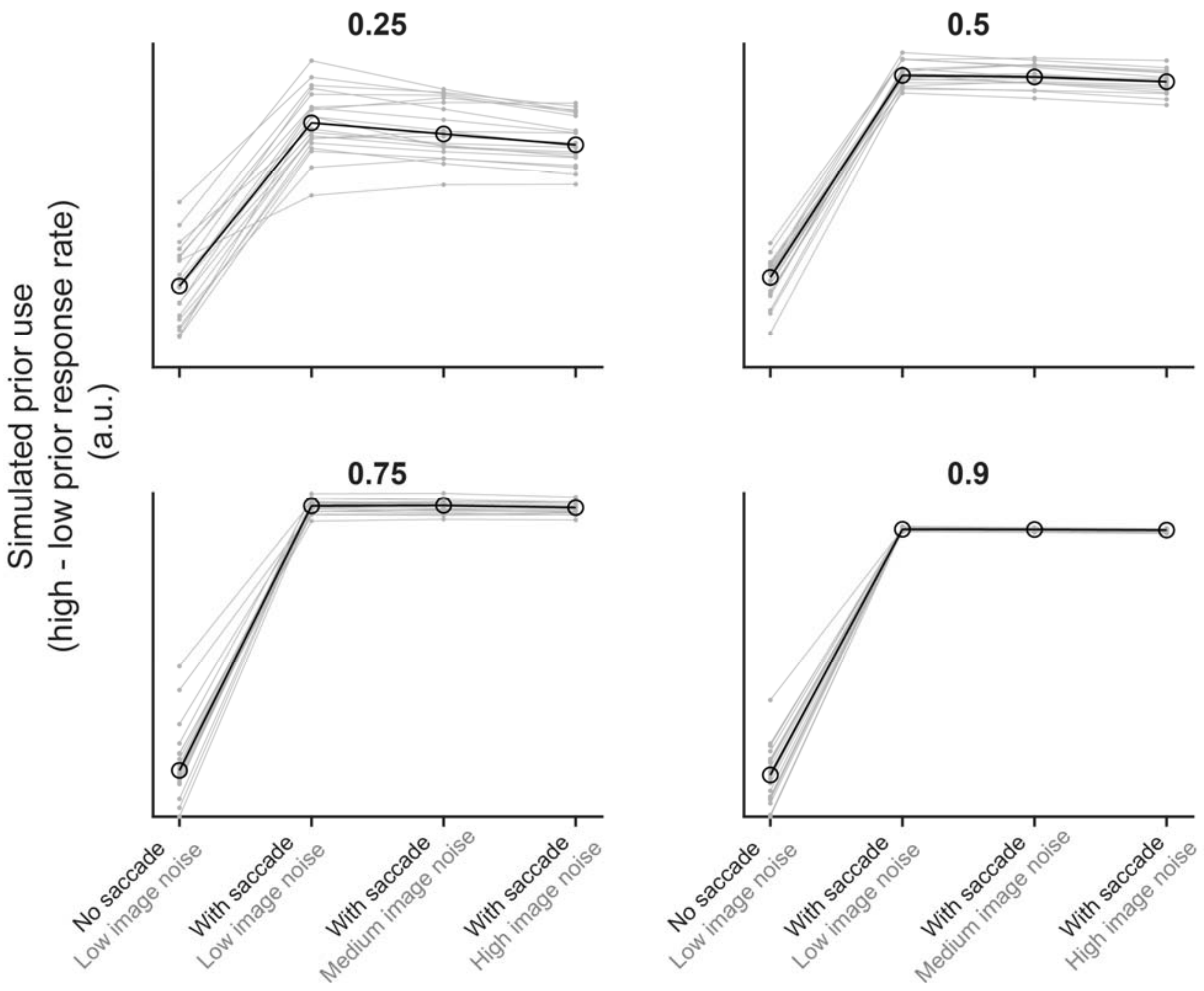

Figure S2. Predicted output of a Bayesian + classifier combined model (same as Fig. 2f) but at four additional weights of the Bayesian component (top left, 0.25; top right, 0. 5; bottom left, 0.75; bottom right, 0.9). Increasing the weight of the Bayesian component has two main effects. First, it modulates the extent to which prior use is influenced by saccade vs. image noise (i.e., slope of the line joining the leftmost two points vs the line joining the three points on the right). For the saccade noise component, this is expected since it is varied in the Bayesian component of the model. For the effect on the image noise conditions where saccade noise is high, the intuition is that the Bayesian component pushes the psychometric curves across prior conditions apart from each other but additional image noise in the categorization component pushes them together. If the weight of the Bayesian component is high enough, it pushes the curve separation to a ceiling such that the dynamic range of the effect of image noise is masked. Note that it does not switch to being a positive slope since image noise is not being varied within the Bayesian component. It reduces the variability in the simulated data. This is because the Bayesian component of the model is deterministic (i.e., for the same input parameter values, it will always produce the same output probability of reporting “jumped”), but the categorization component is not. Since “jumps” and “non-jumps” are drawn from probabilistic distributions and the feedback provided to the perceptron is contingent upon the distribution they were drawn from, it is possible for the same displacement to be classified as either a “jump” or “non-jump” on distinct iterations of the model.

bioRxiv preprint doi: https://doi.org/10.1101/2024.12.31.630950; this version posted April 20, 2026. The copyright holder has placed this preprint (which was not certified by peer review) in the Public Domain. It is no longer restricted by copyright. Anyone can legally share, reuse, remix, or adapt this material for any purpose without crediting the original authors.

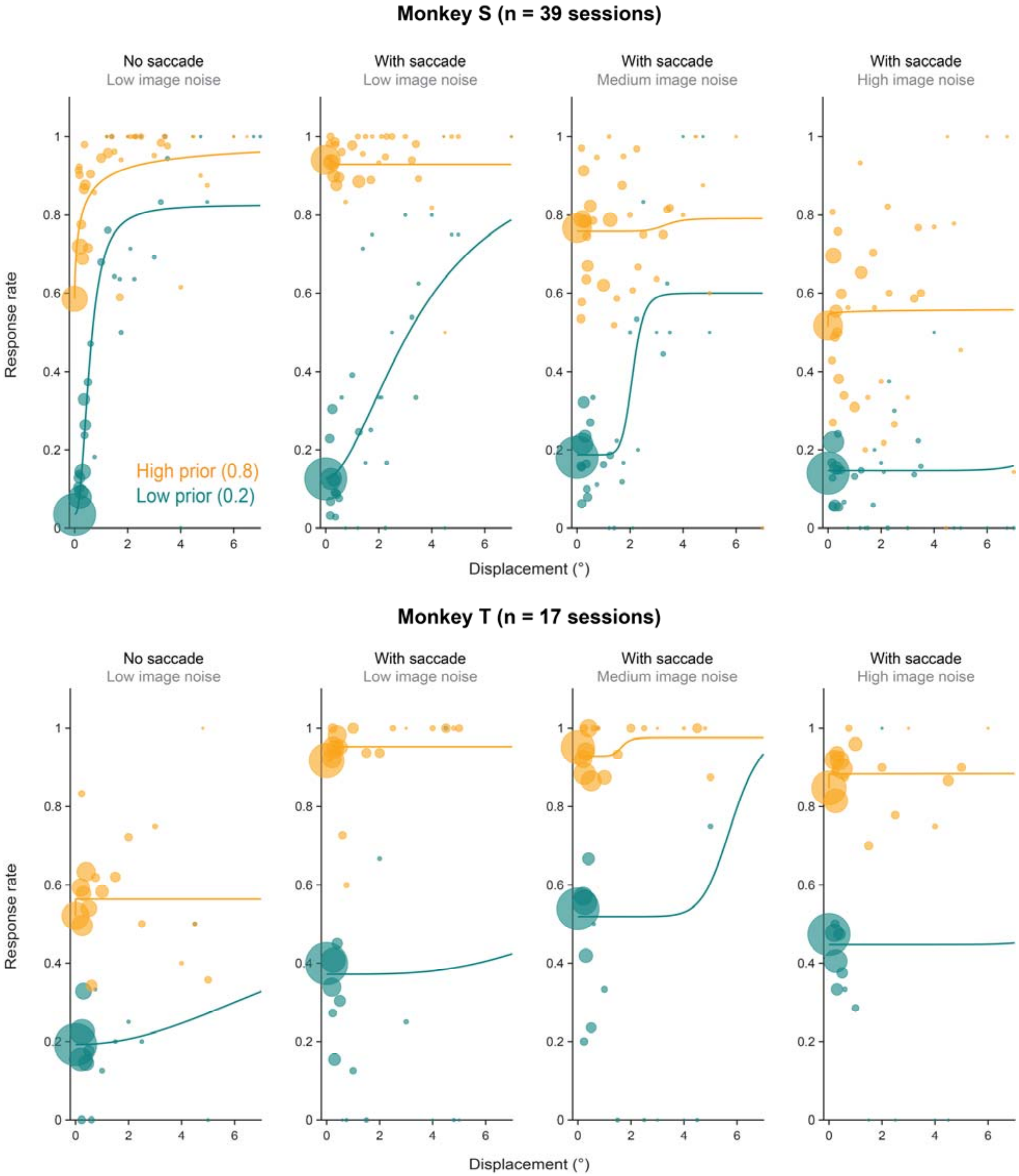

Figure S3. Response rates as a function of displacement across the prior and noise conditions for Monkey S (top) and Monkey T (bottom). Relative sizes of the bubbles indicate the relative numbers of trials at each displacement. Data are pooled across sessions for both animals. Lines show psychometric fits to the pooled data for ease of visualization, but we caution against interpreting their parameters since the spatial configuration and eccentricity of the probe were not held constant across sessions. A comprehensive characterization of

bioRxiv preprint doi: https://doi.org/10.1101/2024.12.31.630950; this version posted April 20, 2026. The copyright holder has placed this preprint (which was not certified by peer review) in the Public Domain. It is no longer restricted by copyright. Anyone can legally share, reuse, remix, or adapt this material for any purpose without crediting the original authors.

psychometric curves for the same animals in versions of the image- and saccade-noise tasks with matched spatial parameters are shown in Subramanian et al. (2023).

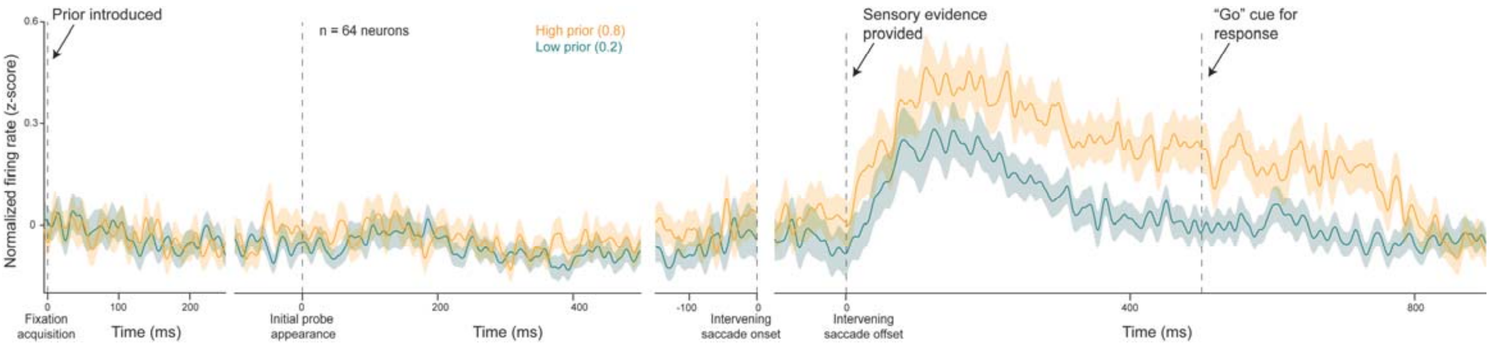

Figure S4. Same as Fig. 3a but restricted to trials where the displacement was 0.

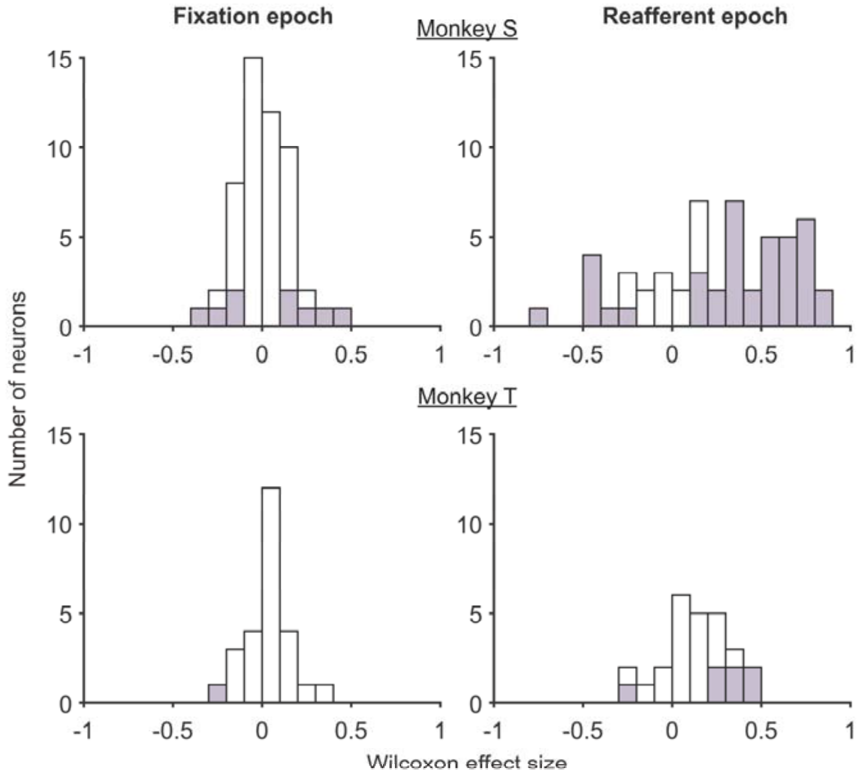

Figure S5. Same as Fig. 3b-c but for each animal individually. Top row: Monkey S, bottom row: Monkey T.

bioRxiv preprint doi: https://doi.org/10.1101/2024.12.31.630950; this version posted April 20, 2026. The copyright holder has placed this preprint (which was not certified by peer review) in the Public Domain. It is no longer restricted by copyright. Anyone can legally share, reuse, remix, or adapt this material for any purpose without crediting the original authors.

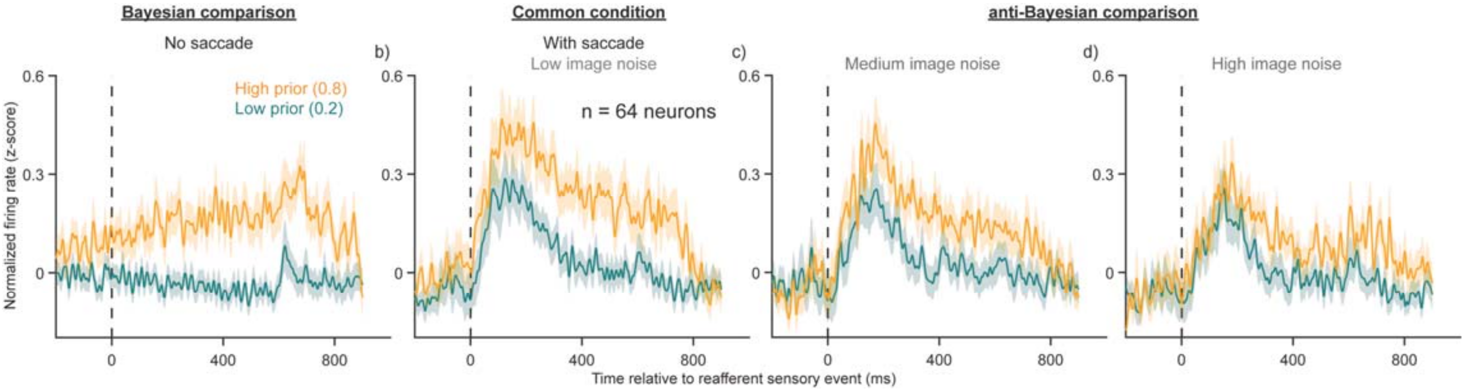

Figure S6. Same as Fig. 5a-d but restricted to trials where the displacement was 0.

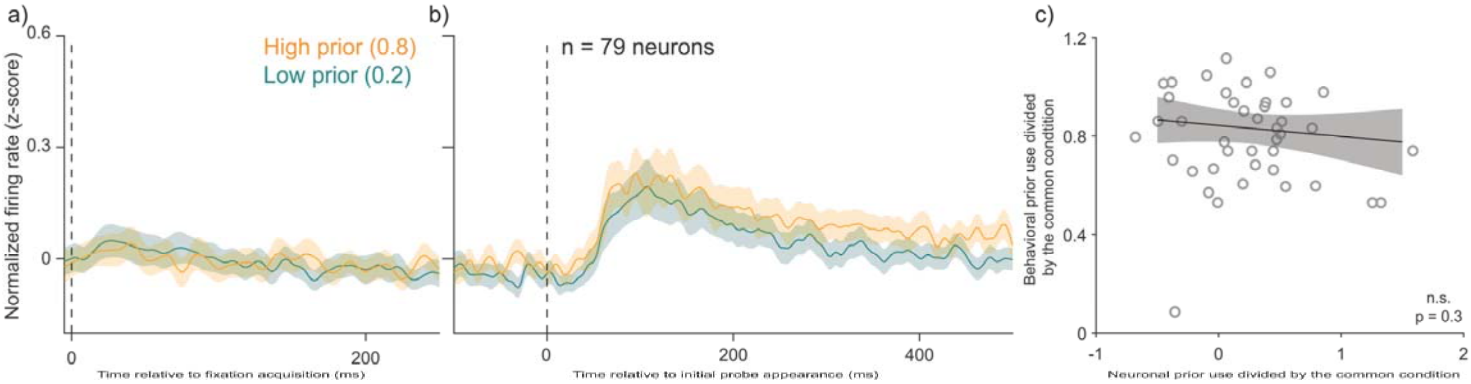

Figure S7. Additional analyses for the no-saccade condition. a-b) Peri-stimulus time histograms in the fixation (a) and probe onset epochs (b) for the no-saccade conditions show overlap in firing rates across the prior conditions. c) There was additionally no correlation between neuronal effect sizes in the probe epoch (0-500 ms from probe appearance) normalized to the common condition and normalized behavioral prior use in the no-saccade, Bayesian comparison condition.

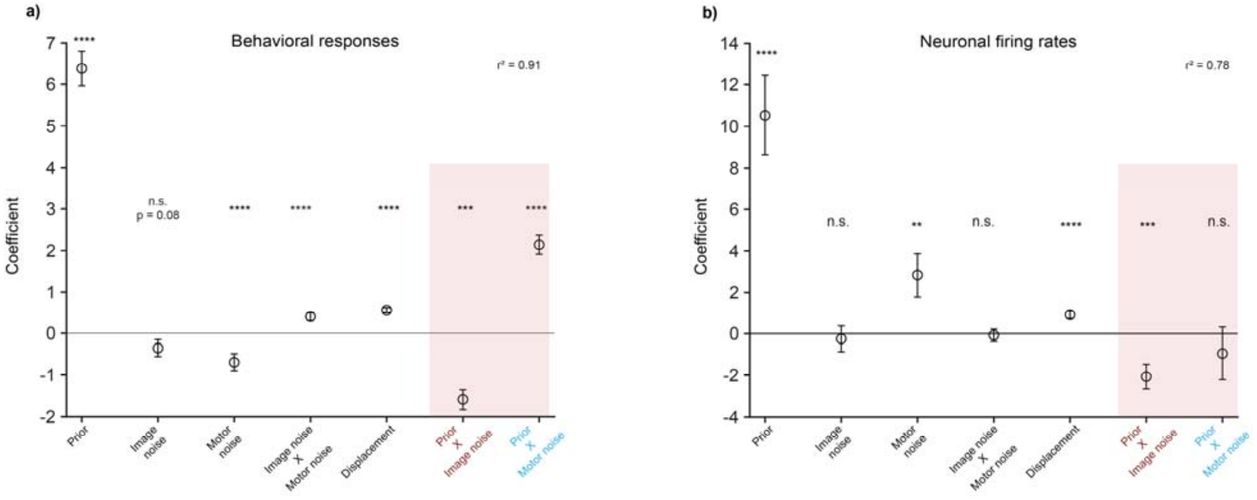

bioRxiv preprint doi: https://doi.org/10.1101/2024.12.31.630950; this version posted April 20, 2026. The copyright holder has placed this preprint (which was not certified by peer review) in the Public Domain. It is no longer restricted by copyright. Anyone can legally share, reuse, remix, or adapt this material for any purpose without crediting the original authors.

Figure S8. Same as Fig. 5i-j but with all neurons (n = 79) included.

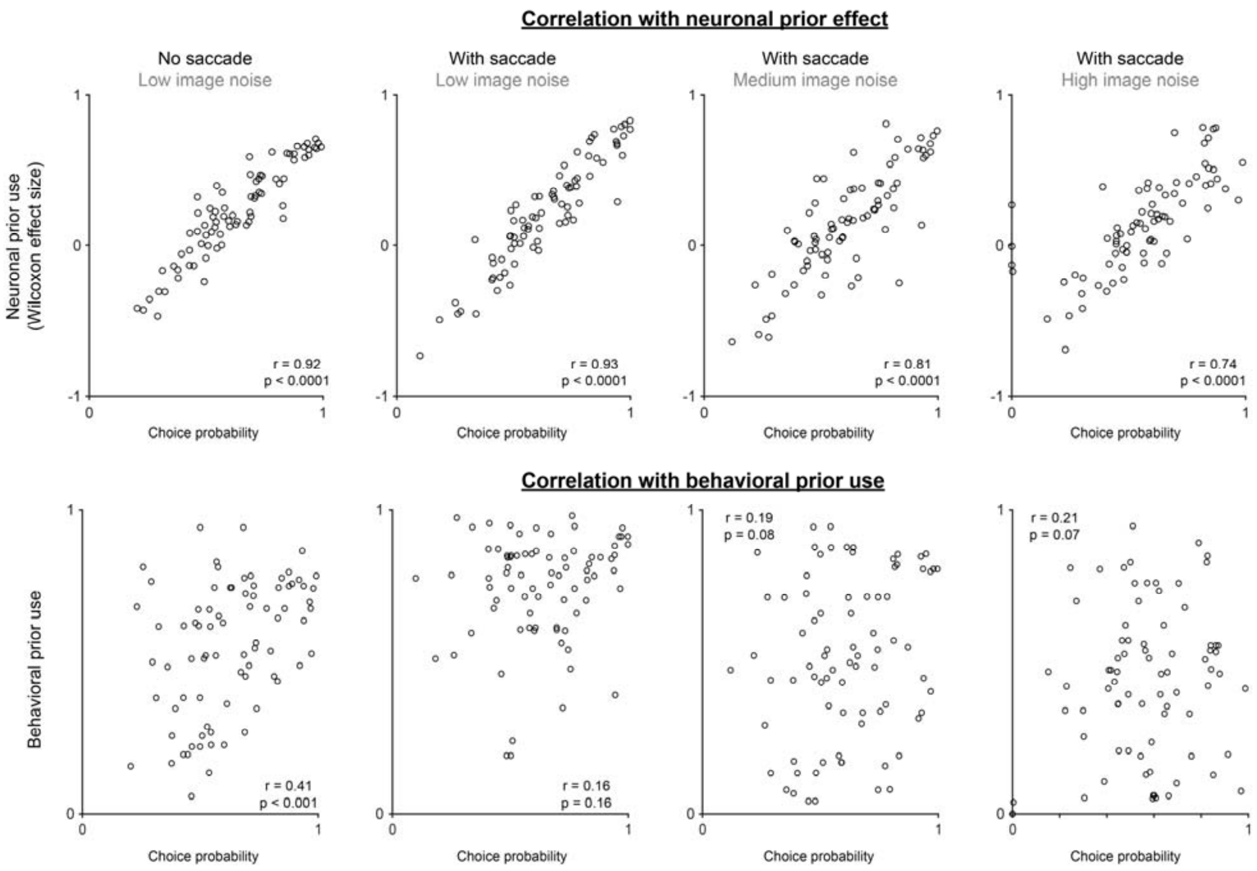

Figure S9. Correlation between choice probabilities for "jumped" vs. "did not jump" responses and prior use. Top row: Correlations with neuronal prior use (Wilcoxon effect size between the high and low prior conditions). Bottom row: Correlations with behavioral prior use (difference in response rates between high and low prior conditions).
